# Supplementary material for: Association of the Vitamin D Level and Quality of School Life in Adolescents with Irritable Bowel Syndrome
Source: J Clin Med. 2018 Dec 1;7(12):500. doi: 10.3390/jcm7120500 (PMC6306771; doi:10.3390/jcm7120500)
Supplement: Supplementary file 1 [file jcm-07-00500-s001.pdf]

Supplementary Data

Figure S1. Questionnaire

Figure S1. Questionnaire

The first question is the Bristol Stool Scale to classify the participants into the irritable bowel syndrome (IBS) subtypes.

Type 1: Small, hard, and lump patterns that look like goat feces

Type 2: Several hard lumps that look like goat feces and form clusters

Type 3: Excrement looks like a sausage but with cracks on its surface

Type 4: Excrement looks like a sausage or snake, smooth and soft

Type 5: Soft blobs with clear-cut edges (passed easily)

Type 6: Fluffy pieces with ragged edges, a mushy stool

Type 7: Watery, no solid pieces, entirely liquid

Second, “How many times did your symptoms such as abdominal pain or bloating disturb you in the last 2 weeks?”

Answer 1: Once a week

Answer 2: 2-3 times a week

Answer 3: 4 times a week

Answer 4: Every day

Third, “How many times do you defecate, regardless of the type and form of stool?”

Answer 1: Once a day

Answer 2: Once or twice a day in IBS with diarrhea (IBS-D)/once in 1-2 days in IBS with constipation (IBS-C)

Answer 3: 3-4 times a day in IBS-D/once in 3-4 days in IBS-C

Answer 4: More than 4 times a day in IBS-D/once in more than 4 days in IBS-C

Fourth, “To what degree do you think symptoms are relieved after defecation?”

Answer 1: Complete relief

Answer 2: Notable symptom relief

Answer 3: Relief to some degree

Fifth, “How many times did your IBS symptoms disturb you in school in the last 2 weeks?”

Answer 1: No effect

Answer 2: Doing well in school but complaining about IBS symptoms

Answer 3: Leaving during school hours or visiting health office because of IBS symptoms

Answer 4: School absence due to IBS symptoms
